# Comparison of postoperative cough-related quality of life and recovery between sublobectomy and lobectomy for early-stage non-small cell lung cancer patients: a longitudinal study

**DOI:** 10.1186/s12890-022-01954-8

**Published:** 2022-04-23

**Authors:** Rongjia Lin, Wen Chen, Leilei Zhu, Xiaojie Pan

**Affiliations:** 1 Department of Thoracic Surgery, Fujian Provincial Hospital, Fuzhou, 350000 People’s Republic of China; 2 The Second Operating Room, Fujian Provincial Hospital, Fuzhou, 350000 People’s Republic of China; 3 Department of Gynecology, Fujian Provincial Maternity and Children’s Hospital, Fuzhou, 350000 People’s Republic of China

**Keywords:** Lung cancer, Enhanced recovery after surgery, Postoperative cough, Leicester Cough Questionnaire

## Abstract

**Background:**

Cough is a common complication after pulmonary surgery. Previous studies lacked a standard measure to assess postoperative cough-related quality of life and recovery. The purpose of this study is to compare postoperative cough regarding changes in health-related quality of life (HRQOL) and recovery trajectory between video-assisted thoracic surgery (VATS) lobectomy and sublobectomy (segmentectomy or wedge resection) for early-stage non-small cell lung cancer (NSCLC) patients via the Leicester Cough Questionnaire in Mandarin Chinese (LCQ-MC).

**Methods:**

Overall, 156 patients with NSCLC underwent either VATS lobectomy or VATS sublobectomy; LCQ-MC was used to report the impact of postoperative cough on HRQOL for 6 months after surgery. The total scores of LCQ-MC range from 3 to 21, with a higher score indicating better health. Recovery from postoperative cough was defined as LCQ-MC scores returning to preoperative levels. The sensitivity of LCQ-MC to changes in postoperative cough recovery over time was evaluated via its ability to distinguish between surgery types.

**Results:**

The VATS sublobectomy group reported significantly higher mean LCQ-MC scores at 1 month after surgery, but no significant difference postoperatively at 3 and 6 months after surgery, and returned to preoperative physical (69 vs. 99 days), psychological (67 vs. 99 days), social (50 vs. 98 days) and total (69 vs. 99 days) scores faster than the VATS lobectomy group (all *p* < 0.05).

**Conclusion:**

VATS sublobectomy had generally better HRQOL and faster recovery of postoperative cough than VATS lobectomy. In addition, the LCQ-MC performed satisfactorily in describing the longitudinal changes in postoperative cough.

## Background

Non-small cell lung cancer (NSCLC) patients who undergo surgery experience an acute systemic inflammatory, neuroendocrine, and metabolic stress response. This response often encompasses a cluster of nonspecific and organ-specific symptoms. Postoperative cough is a common organ-specific symptom that impacts physical, psychologic, and social aspects of daily living; contributes to pain, fatigue, insomnia, and dyspnoea; increases anxiety in patients; and leads to social isolation [1]. Video-assisted thoracic surgery (VATS) is becoming the preferred surgical procedure over open thoracotomy for early-stage NSCLC. The prevalence of postoperative cough varies widely from 39.8 to 46.0% [2–4].

Morbidity and distress levels due to postoperative cough have been underestimated, especially after discharge, representing an unmet clinical need [5]. Recovery from postoperative cough is a complex process that involves physical, psychological, and social domains.

To grasp the potential impact of enhanced recovery after surgery (ERAS) and new operative concepts, the effects of such techniques on a reasonable outcome, such as changes in the status of postoperative cough over time, must first be understood. However, previous studies focused on lobectomy rather than sublobectomy (segmentectomy or wedge resection) and lacked a standard measure to assess recovery from cough symptoms after surgery or discharge. According to the National Comprehensive Cancer Network Clinical Practice Guidelines in Oncology for NSCLC, sublobectomy is appropriate in selected patients with pure adenocarcinoma in situ histology, nodules with a ≥ 50 ground-glass appearance on computed tomography or nodules with a long doubling time (≥ 400 days) confirmed through radiologic surveillance.

We therefore performed a longitudinal study to compare postoperative cough regarding changes in health-related quality of life (HRQOL) and recovery trajectory between VATS lobectomy and sublobectomy for early-stage NSCLC patients. We chose the Leicester Cough Questionnaire in Mandarin-Chinese (LCQ-MC) as the instrument for the investigation and follow-up. This longitudinal study was performed in accordance with the STROCSS Reporting Checklist [6].

## Methods

### Subjects

A total of 166 patients who underwent VATS performed by a single medical team between May 2019 and October 2020 at the Department of Thoracic Surgery, Fujian Provincial Hospital were enrolled. Patients were included according to the following criteria: (1) aged 18 years or older, (2) had postoperative cough but no cough symptoms within the 2 weeks prior to surgery, (3) provided a signed informed consent form, (4) presented with resectable TNM stage I lung cancer, (5) underwent VATS, and (6) had postoperative pathological findings indicative of NSCLC. Patients were excluded according to the following criteria: (1) underwent bilateral pulmonary surgery, (2) underwent conversion to open thoracotomy or experienced bleeding exceeding 1000 mL, (3) were transferred to the intensive care unit after surgery, or (4) refused to answer the survey or dropped out.

### Surgical procedures

Single utility port VATS lobectomy or sublobectomy was performed [7]. Systematic mediastinal lymphadenectomy was performed in accordance with the Chinese guidelines for the diagnosis and treatment of primary lung cancer (2015) [8]. Sublobectomy achieved parenchymal resection margins ≥ 2 cm or ≥ the size of the node and sampled the appropriate N1 and N2 lymph node stations. Mediastinal lymphadenectomy included stations 2R, 4R, and 7–9 for right-sided cancer and 4Land 5–9 for left-sided cancer [9]. Lymph node dissection included the N1 and N2 nodes with a minimum of 3 N2 stations sampled or complete dissection [10].

A 30 Fr chest tube was placed in the observation port for postoperative drainage (7th or 8th intercostal space), while a 22 or 30 Fr chest tube was placed in the 2nd intercostal space at the midclavicular line (usually upper lobectomy).

The patients were encouraged to engage in ambulation soon after surgery, and a chest X-ray was scheduled for postoperative day (POD) 1. The chest tube withdrawal criteria included the absence of air leakage through the chest tube, a pleural fluid drainage of < 200 mL in 24 h, and no pneumothorax, haemothorax or chylothorax. The chest tubes were removed as early as possible on POD 1.

### Assessment measurements and endpoint

The LCQ-MC, which assesses the impact of cough on HRQOL, consists of 19 items divided into three domains: physical (8 items), psychological (7 items), and social (4 items). A 7-point Likert scale was used to score the individual domains. The total scores ranged from 3 to 21, with a higher score indicating better health [11, 12]. We previously showed that the LCQ-MC reliably assessed postoperative cough in patients with NSCLC who underwent VATS [13].Patients completed the LCQ-MC before discharge and at multiple timepoints during the 6 months after surgery.

We defined “recovery from postoperative cough” when the patients reported three domain score or a total LCQ-MC score that had returned to the preoperative (baseline) level. We conducted the LCQ-MC on eligible patients before discharge, 1 month, 3 months, and 6 months after surgery. The follow-up endpoint was indicated if the cough symptoms disappeared during this period.

Postoperative diagnoses were determined using the 2015 World Health Organization Classification of Lung Tumours and the eighth edition of the Union for International Cancer Control /American Joint Committee on Cancer lung cancer staging classification guidelines [14, 15]. Regional lymph node classification was based on the International Association for the Study of Lung Cancer lymph node map [16].

### Data collection

Inpatients completed paper questionnaires while in the wards with the assistance of our investigators before discharge. Postoperative follow-up was performed by telephone or outpatient review at 1, 3, and 6 months after surgery. All data were uploaded to a network database for management and analysis (jinshuju, a data collection and management platform, https://jinshuju.net/).

### Statistical analysis

Patient characteristics are presented as the mean ± standard deviation (SD) for continuous variables and as relative frequencies for categorical variables. We performed t-tests, Pearson’s χ^2^ tests and Yates’s correction for continuity, as appropriate, to identify differences between the lobectomy and sublobectomy groups. All comparisons were 2-sided, and differences with *p* < 0.05 were considered statistically significant. Recovery duration from each surgery type was estimated for each interference item using Kaplan–Meier analysis, and log-rank tests were used to compare time to recovery by surgery type. Statistical analyses were performed using IBM SPSS Statistics, version 23.0 (Statistical Package for the Social Sciences, Chicago, IL, USA) and GraphPad Prism, version 9.0 (GraphPad Software, San Diego, CA, USA).

## Results

### Patient characteristics

A total of 166 patients consented to participate in the study, and 156 patients completed follow-up (attrition rate 6.02%, 10/166 patients). 8 patients withdrew during the follow-up period (2 in month 1, 5 in month 3, and 1 after month 6), and 2 patients underwent bilateral pulmonary surgery. Approximately 42% (65/156) of the patients underwent VATS sublobectomy, and there was no significant difference in the baseline data between two surgery types. Table 1 shows the baseline demographic and clinical characteristics of the patients.

**Table 1:** Clinical characteristics and demographic

	Lobectomy(n = 91)	Sublobectomy(n = 65)	*P* value
Age, years	59.82 ± 10.01	57.08 ± 11.17	0.111
Sex, *n* (%)			
Male	31(34.1)	23(35.4)	0.499
Female	60(65.9)	42(64.6)	
Smoking history, *n* (%)			
Yes	20(22.0)	13(20.0)	0.463
No	71(78.0)	52(80.0)	
Lung function			
FEV1(L)	2.47 ± 0.55	2.50 ± 0.53	0.816
FVC(L)	3.04 ± 0.67	3.26 ± 0.66	0.144
Operated side			
Right	51(56.0)	34(52.3)	0.382
Left	40(44.0)	31(47.7)	
Pathological diagnosis, *n* (%)			
Adenocarcinoma	85(93.4)	62(95.4)	0.418
Squamous carcinoma	6(6.6)	3(4.6)	

Data are presented as mean ± SD or *n* (%)

### Longitudinal profiles of postoperative cough

The median time to the onset of cough symptom after VATS lobectomy was 1 day (interquartile range [IQR], 0–2), compared with VATS sublobectomy was 2 days (IQR, 1–2). The median duration of postoperative cough in patients who underwent VATS lobectomy was 98 days (IQR, 39–155), and 15 patients (15/91, 16.5%) still had cough symptom at 6 months after surgery. The median duration in the VATS sublobectomy group was 65 days (IQR, 35–107), and 5 patients (5/65, 7.7%) had cough symptoms at 6 months after surgery.

**Fig. 1:**
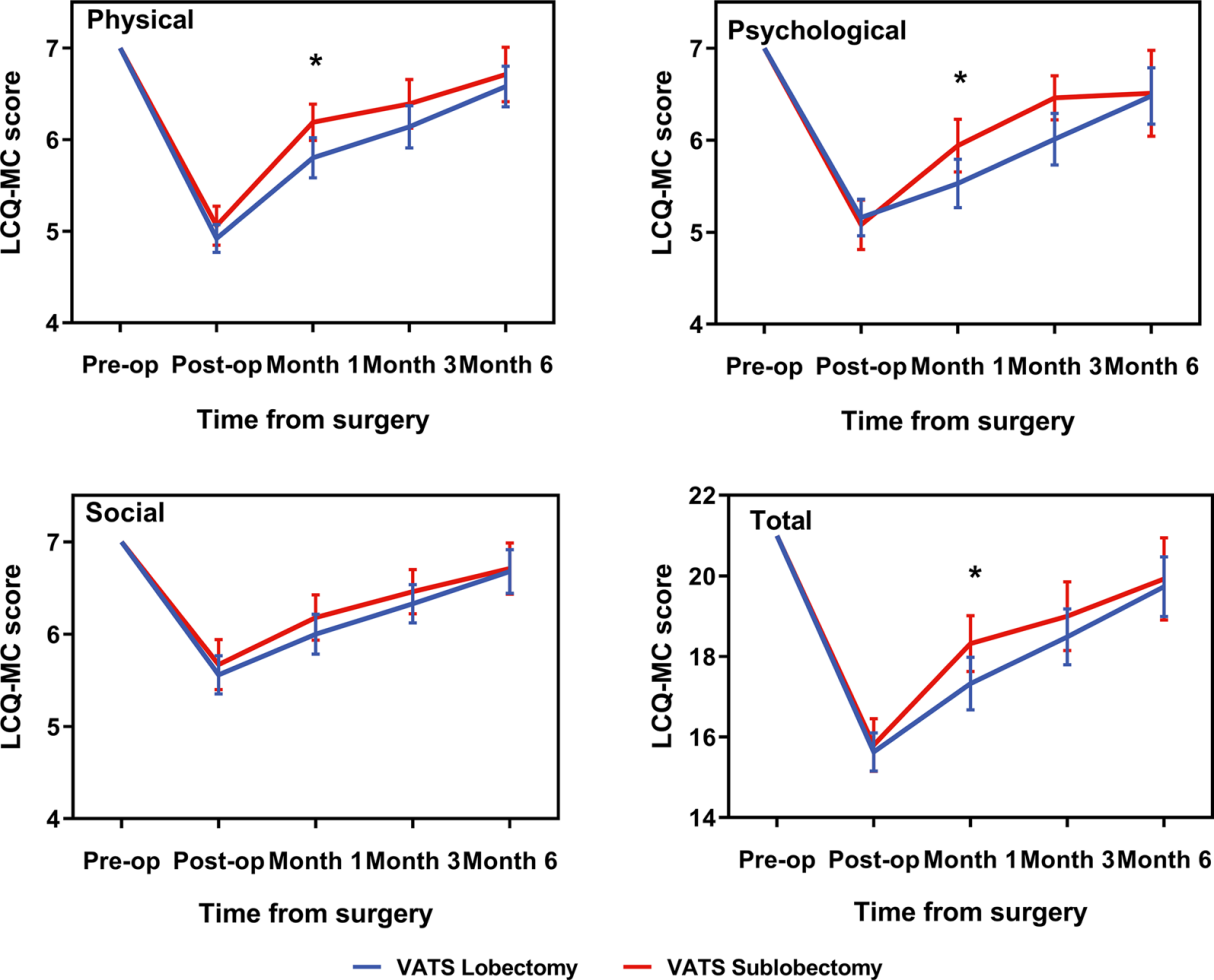
Change in average LCQ-MC scores during the 6 months after surgery, by surgery type. *p < 0.05. LCQ-MC, Leicester Cough Questionnaire in Mandarin-Chinese; VATS, video-assisted thoracic surgery

Figure 1 shows that the individual domain score and total LCQ-MC score decreased rapidly after surgery and then returned to preoperative levels over time. The VATS sublobectomy group reported significantly higher mean physical, psychological, and total LCQ-MC scores than the VATS lobectomy group at 1 month after surgery (all *p* < 0.05). However, the mean LCQ-MC scores of the VATS sublobectomy group were higher than those of the VATS lobectomy group at postoperative 3 and 6 months after surgery, but the differences were not significant. The dynamic changes and detailed scores are shown in Fig. 1 and Table 2.

**Table 2:** The mean LCQ-MC scores after surgery and follow-up

	Lobectomy (n = 91)	Sublobectomy (n = 65)	*P* value
Postoperative LCQ-MC			
Physical	4.92 ± 0.73	5.06 ± 0.86	0.268
Psychological	5.16 ± 0.95	5.08 ± 1.08	0.643
Social	5.56 ± 1.00	5.67 ± 1.10	0.516
Total	15.63 ± 2.27	15.80 ± 2.65	0.657
LCQ-MC at 1 month			
Physical	5.80 ± 1.05	6.19 ± 0.80	0.014
Psychological	5.53 ± 1.26	5.94 ± 1.16	0.038
Social	6.00 ± 1.04	6.18 ± 0.99	0.257
Total	17.33 ± 3.14	18.32 ± 2.82	0.045

	Lobectomy(n = 67)	Sublobectomy (n = 41)	*P* value
LCQ-MC at 3 months			
Physical	6.14 ± 0.95	6.39 ± 0.81	0.160
Psychological	6.01 ± 1.15	6.16 ± 1.17	0.561
Social	6.33 ± 0.85	6.46 ± 0.73	0.426
Total	18.49 ± 2.83	19.00 ± 2.61	0.356

	Lobectomy(n = 36)	Sublobectomy (n = 22)	*P* value
LCQ-MC at 6 months			
Physical	6.58 ± 0.66	6.71 ± 0.58	0.472
Psychological	6.48 ± 0.91	6.51 ± 0.91	0.933
Social	6.68 ± 0.70	6.71 ± 0.54	0.876
Total	19.74 ± 2.19	19.93 ± 1.98	0.766

Data are presented as mean ± SD

### Recovery time from postoperative cough as measured by the LCQ-MC

**Fig. 2:**
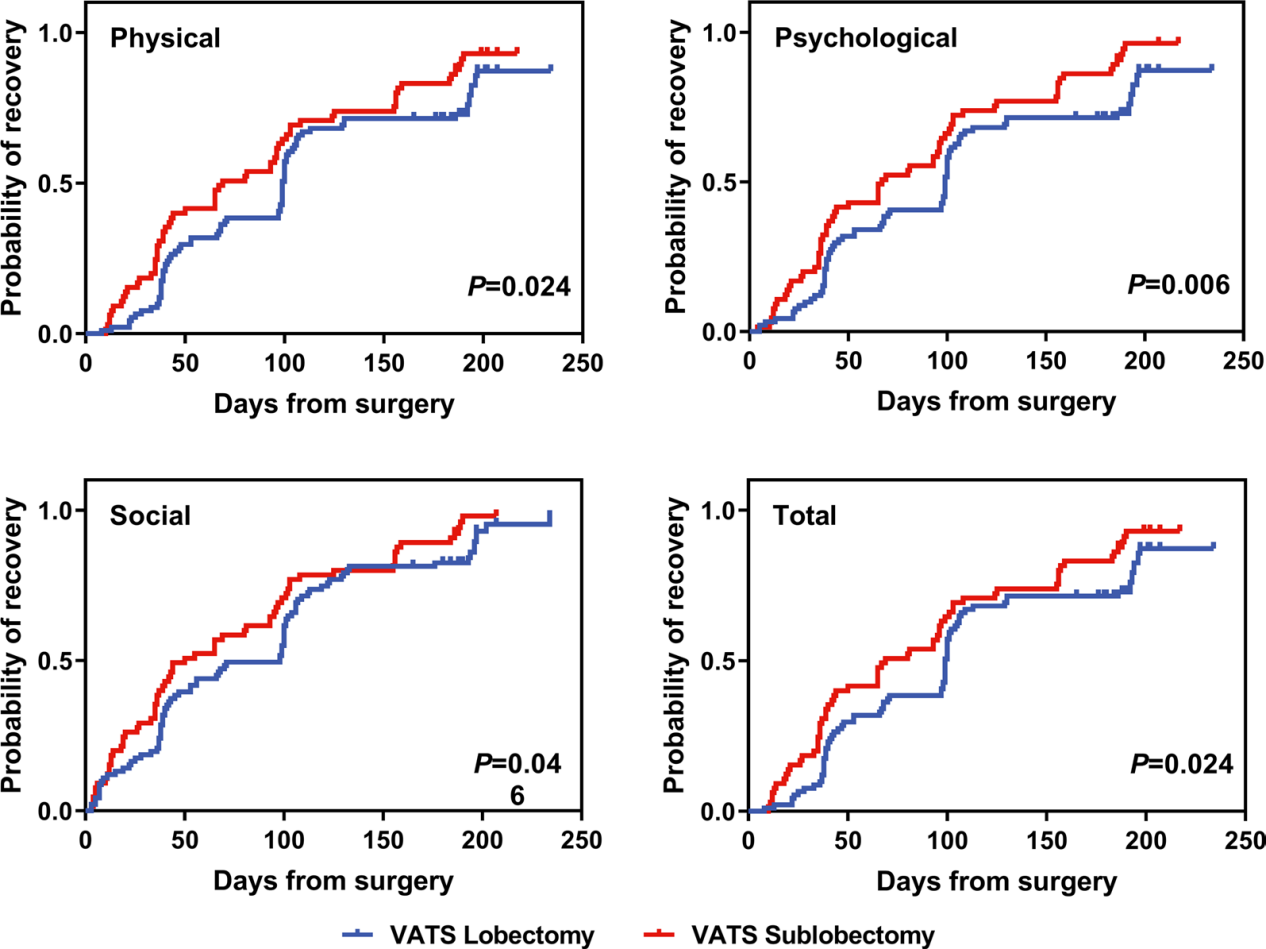
The recovery trajectories of postoperative cough by surgery type. *P* value by log-rank test. VATS, video-assisted thoracic surgery

Table 3 shows that the physical, psychological, and total LCQ-MC scores returned to preoperative levels for all patients at approximately 3 months. The social score of the LCQ-MC took only 2 months recover. Figure 2 presents the profiles of and significant differences in the physical, psychological, social, and total aspects recovery from postoperative cough between VATS lobectomy and VATS sublobectomy by Kaplan–Meier analysis, confirming the sensitivity of LCQ-MC for indicating recovery after VATS. Compared with patients who underwent VATS lobectomy, patients who underwent VATS sublobectomy needed significantly less time to return to baseline levels of the physical aspect (69 vs. 99 days, *p* = 0.024), psychological aspect (67 vs. 99 days, *p* = 0.006), social aspect (50 vs. 98 days, *p* = 0.046) and total aspect (69 vs. 99 days, *p* = 0.024) of postoperative cough (Table 3).

**Table 3:** Kaplan–Meier estimated postoperative cough recovery time (days from surgery)

LCQ-MC	Median days to recovery (95% confidence interval)^a^			*P*
	Overall	Lobectomy	Sublobectomy	
Physical	97(85–109)	99(98–100)	69(35–103)	0.024
Psychological	96(78–114)	99(97–101)	67(36–98)	0.006
Social	66(37–95)	98(72–124)	50(27–73)	0.046
Total	97(85–109)	99(98–100)	69(35–103)	0.024

Data are presented as median(95%CI)

^a^Recovery of postoperative cough was defined as the patient having reported LCQ-MC individual domain or total score at the preoperative (baseline) level

## Discussion

This longitudinal study is the first to specifically compare postoperative cough regarding changes in HRQOL and recovery trajectory between VATS lobectomy and sublobectomy for early-stage NSCLC patients. In addition, this study demonstrates the potential utility of the LCQ-MC, a cough-specific instrument to assess impact on HRQOL [17]. To the best of our knowledge, this study is the first to define the recovery from postoperative cough using the LCQ-MC as an outcome measure and compare the recovery between VATS lobectomy and VATS sublobectomy.

Sublobectomy is fundamentally a more limited parenchymal resection and is being advocated for early lung cancer. The advantages of sublobectomy could be preservation of lung function, low frequency of surgical morbidity and mortality, less intraoperative blood loss, shorter hospital stay, and so on [18]. As expected, VATS sublobectomy had more favourable effects on postoperative recovery and morbidity than VATS lobectomy [19, 20]. This may be related to a combination of factors associated with VATS lobectomy, such as greater stress on the cardiovascular system, larger change in the bronchial angle and more stimulation to cough receptors [21–23]. Postoperative cough, one of the most common respiratory symptoms after surgery, can adversely affect HRQOL [24]. In severe postoperative cough, patients experience disrupted sleep and difficulty talking, which increases their psychological burden and worsens their HRQOL [2, 25, 26].

The LCQ-MC enables real-time reporting of the impact of postoperative cough on HRQOL in terms of physical, psychological and social domains to the surgeon or health care provider, which may improve the likelihood of effectively evaluating ERAS [27]. Patient satisfaction after surgery is largely dependent on his or her recovery experience [28]. Accurate and convenient evaluations of the impact of postoperative cough on HRQOL are important for ensuring early and effective treatment [29].

Postoperative cough peaked immediately after surgery and were prevalent on PODs 1–2, representing a combined effect from surgical trauma and perioperative care. The patients who underwent VATS sublobectomy reported significantly higher physical, psychological, and total LCQ-MC scores at 1 month after surgery than those who underwent VATS lobectomy. Our finding of the different impacts of postoperative cough on HRQOL between the two groups, as measured by LCQ-MC, mirrors previous studies that have reported that the severity of postoperative cough in early-stage NSCLC patients after VATS sublobectomy was lower or similar to that in patients after VATS lobectomy [27, 30, 31]. In patients who underwent VATS sublobectomy, the postoperative cough returned to preoperative levels after approximately 2 months (median time for the physical score: 69 days, psychological score: 67 days, and social score: 50 days). In patients who underwent VATS lobectomy, the postoperative cough had a somewhat different pattern of recovery in this study and recovered more slowly (median time for the physical score: 99 days, psychological score: 99 days, and social score: 98 days) (Fig. 2; Table 3).

Using LCQ-MC as an indicator of the status of postoperative cough symptoms, we not only defined the impact of postoperative cough on HRQOL in this study but also defined the time course of postoperative cough recovery after VATS for early-stage NSCLC. In the current study, repeated LCQ-MC measurements were acquired at selected critical timepoints, beginning with a preoperative assessment, thus sufficiently and sensitively capturing significant differences in the impact of postoperative cough on HRQOL over time and the time needed to return to preoperative levels between two different surgery types for early-stage NSCLC. The minimal clinically important difference (MCID) is the smallest change in the quality-of-life score considered to be clinically meaningful [32]. The mean (SD) total LCQ MCID was 1.3 (3.2), the MCIDs for each domain were as follows: physical: 0.2 (0.8), social: 0.2 (1.1) and psychological: 0.8 (1.5) [33]. In the current study, the differences between the immediately postoperative individual physical, psychological, and social domain scores as well as total LCQ-MC scores and those acquired 1 month after surgery were 0.96 (0.96), 0.53 (1.29), 0.43 (1.12) and 1.92 (2.99), respectively, suggesting a clinically meaningful difference.

This study had several limitations. First, the sample size was imbalanced in two groups and insufficient to control potentially relevant risk factors. Future studies with a more diverse sample are warranted to identify potential risk factors by multivariate analysis and define the trajectory of postoperative cough recovery to increase the generalizability of the results. Second, we usually administered the LCQ-MC on the day of discharge and did not collect the LCQ-MC scores on PODs 1–3 or 1 week after surgery. And postoperative follow-up should be performed monthly, instead of 1, 3 and 6 months after surgery. The differences in postoperative cough between VATS lobectomy and VATS sublobectomy that were observed clinically may have been even more striking if these data had been included in the analysis. Third, in addition to charting the return to preoperative levels, the study lacked another objective measure of cough by which to define recovery to a “good” level of postoperative cough (both cut-off points and MCID), which could be more practical for further decision-making about ongoing cough-specific treatment, and there would be no need for a baseline LCQ-MC assessment.

In conclusion, this study is the first to describe the nature of and changes in postoperative cough between sublobectomy and lobectomy for early-stage NSCLC patients. VATS sublobectomy had generally better HRQOL and faster recovery of postoperative cough than VATS lobectomy. In addition, the LCQ-MC is a clinically relevant and user-friendly instrument for assessing the patient’s perspective of the impact of postoperative cough on HRQOL and monitoring its recovery trajectory.

## Data Availability

This is part of a larger study. The dataset generated and analyzed during the current study is not publicly available but may be obtained from the corresponding author if accompanied by a reasonable request.

## Abbreviations

HRQOL: Health-related quality of life
LCQ-MC: Leicester Cough Questionnaire in Mandarin-Chinese
NSCLC: Early-stage non-small cell lung cancer
VATS: Video-assisted thoracic surgery
ERAS: Enhanced recovery after surgery
POD: Postoperative day
SD: Standard deviation
IQR: Interquartile range
MCID: Minimal clinically important difference
